# Buffalo City Hospital

**Published:** 1846-12

**Authors:** 


*Buffalo City Hospital.*—The building known as the "Seaman's Home" has been obtained, temporarily, to be used as a City Hospital, until other and more extensive accommodations can be procured.

